# Does Raising Livestock Improve Household Food Security and Child Dietary Diversity in a Rural Region of Madagascar?

**DOI:** 10.3390/children10050765

**Published:** 2023-04-23

**Authors:** Fanantenana Raholiarimanana, Hasina Rakotomanana, Akira Ishida

**Affiliations:** 1 Graduate School of Agricultural Science, Kobe University, Kobe 657-8501, Japan; 2 Department of Nutritional Sciences, Oklahoma State University, Stillwater, OK 74078, USA

**Keywords:** livestock, food security, dietary diversity score, Madagascar, children, complementary feeding, Household Food Insecurity Access Scale

## Abstract

Madagascar is one of the poorest countries and has an alarming prevalence of food insecurity and child undernutrition. Most of the Malagasy population live from agricultural activities making livestock a livelihood asset and a source of animal-source foods, especially for smallholder farmers. This study aimed to examine the association between livestock ownership, household food security, and children’s dietary diversity in a rural region of Madagascar. Data from a cross-sectional survey of 344 respondents were used to assess the association between household tropical livestock units (TLU) per capita, Household Food Insecurity Access Scale (HFIAS) scores, and dietary diversity scores (DDSs) among children aged 6–23 months. The estimation results from the ordered probit model showed that household TLU per capita is negatively associated with HFIAS scores and positively associated with DDSs among children. Additionally, households with mothers who received information on childcare and nutrition from health facilities and community nutrition agents were more likely to be food secure and have better dietary diversity. Therefore, promoting livestock ownership and strengthening nutrition-sensitive messages focusing on the benefits of raising livestock to mothers from rural Madagascar will likely be effective in improving household food security and nutrition for children.

## 1. Introduction

Agriculture is one of the main activities sustaining smallholder farmers’ livelihoods in low-income countries and provides employment for two thirds of the African working population, allowing them to access nutritious foods [1]. However, due to their usually poor agricultural performance, smallholder farmers are often vulnerable to hunger [2]. Millions of people suffer from undernourishment and malnutrition, principally in Sub-Saharan Africa, where one in three people experiences chronic hunger [3].

In such contexts, livestock are an asset to help support improvements in the rural economy and livelihood and alleviating food shortages [4]. Livestock are the principal source for animal-based foods, and their benefits on household food security and diet quality improvement has been reported in many low- and lower-middle-income countries [5,6,7,8,9,10,11]. Additionally, agricultural and livestock interventions improved vulnerable households, women’s, and children’s dietary diversity in Zimbabwe [10], Mali [12], Ethiopia [11], and Kenya [13]. Children fed with more diversified diets are more likely to meet their nutrient requirements [14,15,16] for optimal physical growth [17] and development [18,19,20].

Moreover, livestock have been shown to play a key role in poverty reduction as they contribute to improving rural incomes [6]. Integrated with crop production, livestock farming can ensure the enhancement and stability of rural livelihoods [21]. Hatab et al. [22] and the World Bank [23] pointed out that livestock production leads to increased accessibility of preferred or high-quality nutritious food by improving farm incomes for vulnerable households, particularly in remote areas. Furthermore, livestock remain one of the main assets of smallholders, as they constitute both a financial and a social capital making it possible to sustain livelihoods [4]. 

Despite these well-known benefits, the effects of livestock rearing on household food security and dietary diversity among children have not been investigated in Madagascar. The country is far from meeting the global nutrition targets for 2025 [24] or fulfilling its commitment to the Sustainable Development Goals of tackling undernutrition and ending hunger by 2030 [25]. Madagascar is still coping with a high burden of child undernutrition and high levels of household food insecurity. Between 2019 and 2021, 48.5% of Malagasy people were undernourished, 10.3% were severely food-insecure, and 61.1% were moderately food-insecure [1]. While much progress has been made since 2012 regarding the prevalence of stunting among children under five, it was still at an alarming level of 42% in 2018 [25]. Due to recurrent economic crises, climate shocks, and political instability, Madagascar is affected by chronic poverty and frequent food crises [26]. Approximately 81.5% of Malagasy people were living on less than USD 2.15 per day in 2021, and 63.9% of the local population relies on agriculture for subsistence [27].

Many studies have investigated the principal determinants of child undernutrition and food insecurity in Madagascar [28,29,30,31,32,33,34,35,36,37,38]. However, the links between livestock rearing, household food security, and children’s dietary diversity are not entirely understood. Very few studies including an initial study in the Moramanga and Morondava regions of Madagascar [9] showed a positive association between livestock ownership and high dietary diversity scores (DDSs). 

Therefore, this study aimed to examine the relationship between livestock ownership, household food security status, and children’s dietary profiles in the region of Vakinankaratra, one of its most agriculturally productive regions with thriving livestock farming. Results of this study can shed light on the importance of livestock rearing for improving food security and child nutrition in an agricultural region of the country.

## 2. Materials and Methods

### 2.1. Study Setting and Sampling Method

A cross-sectional survey was conducted in the Vakinankaratra region in April 2019 to assess household food insecurity and children’s diet quality. Vakinankaratra is the most populated region after the capital and is located in the central highlands of Madagascar. Known as one of the three largest agricultural regions in Madagascar, the Vakinankaratra region provides a large amount of the food supply for the entire country. The region is well known for cattle breeding for dairy production, as it is one of the poles of the milk triangle in supplying milk to local consumers and dairy industries in central Madagascar. The region has the highest level of stunting (60%) in the country [25] and only 32.2% of children under five have reached the minimum dietary diversity in the region [25].

The region is mainly rural and includes seven districts divided into 39 communes, which are in turn subdivided into fokontany or villages. Most villages have community nutrition centers (CNCs) where mothers receive nutrition counseling on feeding practices from the community nutrition agents (CNAs). Growth monitoring activities for children are also conducted in CNCs. A multi-stage cluster sampling method was used for this study. In the first stage, the districts of Antanifotsy and Antsirabe II were selected, omitting the districts of Mandoto and Faratsiho because of the difficult access, as well as the district of Antsirabe I, an urban district [39]. The second stage consisted of selecting communes in the two identified districts according to the density of their populations. Nine communes were selected: three from the district of Antanifotsy and the rest from the district of Antsirabe II. For the selection of fokontany, those with a community nutrition center accessible by car or, at most, by two hours of walking were considered during the selection. A random selection of five fokontany per commune was performed, resulting in a total of 43 fokontany.

Mothers with children 6–23 months old who were married or living with their partner were eligible for the study. The community nutrition agents established a list of mothers who regularly frequented the community nutrition centers, and a random selection was carried out to select ten mothers per fokontany. Therefore, 391 mothers were considered for the survey, using a margin of error of 5%, a Z score for a level of confidence of 95% equal to 1.96, and a prevalence of stunting of 55% from a 2017 survey [40]. Approximately 96.2% and 81.1% of the surveyed mothers responded that their households possessed agricultural land and livestock, respectively, for farming. Ultimately, 344 respondents were retained for empirical analysis after refining the dataset and eliminating individuals with missing or inconsistent data.

### 2.2. Questionnaire

Surveys were conducted with mothers using a pretested questionnaire. Data on the socioeconomic and demographic characteristics of the household; the characteristics of the mothers and their children; maternal knowledge regarding nutrition, breastfeeding, and complementary feeding practices; household food insecurity experience; and fathers’ involvement in childcare were obtained from the survey. The sources of the nutrition information possessed by the mothers were also collected. For the complementary feeding section, information regarding breastfeeding, food consumption, and meal frequency was included. The section on the father’s involvement included paternal role in providing financial support for children’s food and their participation in different activities related to childcare.

A section related to household food security was added to evaluate the Household Food Insecurity Access Scale (HFIAS) score for each household. This indicator measures a household’s experiences when constrained by food inaccessibility and when the family has to compromise on food quantity or quality due to periodical lack of resources within the previous days [41]. The HFIAS is widely used in food insecurity assessments and it employs nine items regarding food insecurity with scores ranging from 0 to 3 for each question according to the severity of the food inaccessibility experience. The final total score is calculated by summing each individual question scores, and ranges from 0 to 27. Higher scores indicate higher levels of food insecurity [41].

WHO guidelines were used to assess infant and young child complementary feeding indicators, including dietary diversity. During data collection, the WHO 2008 guidelines were used [42,43], but the analyses were based on the 2021 guidelines [44]. The only difference in the data used is that, in the revised guidelines, breast milk is counted as a separate food group. Of the eight defined food groups, including breast milk, the child must have consumed at least five during the previous day to achieve the minimum dietary diversity [44]. Using the 24 h recall method, a score in the range of 0 to 8 for the dietary diversity was deduced from the sum of all food groups consumed by the child [45,46]. 

The number of livestock animals for each household, including numerical variables for bulls, cows, pigs, poultry, goats, lambs, and rabbits, was transformed to tropical livestock units (TLU). The TLU indicator is a common unit that represents the total size of a household’s livestock holdings. Different studies have used the TLU as a measurement unit for livestock ownership to explore a wide variety of factors, including food security [47,48]. According to Jahnke [49] and Rothman-Ostrow et al. [50], the weight of each species should be calculated based on its biomass using the Sub-Saharan Africa standard of animal weight measures.

TLU per household was calculated according to the following formula:

TLU = bull/1.43 + cow/1.43 + pig/5 + lamb/10 + poultry/100 + rabbit/100,(1)

Considering the disparity in household size, household TLU per capita [34] was used in the analysis by dividing the household TLU by the size of the household.

### 2.3. Empirical Approach

To examine the associations between TLU per capita and the HFIAS and DDS, a bivariate ordered probit model was first applied on the assumption that the error terms of the two equations were correlated. If the error terms of the two equations were not correlated significantly, the parameters of the two equations would not need to be estimated simultaneously by applying the bivariate ordered probit model but separately by using the ordered probit model for each equation.

To confirm the strength of the association of TLU per capita with the HFIAS and DDS, other independent variables were added to the final model of each equation. In addition to variables denoting the attributes of the surveyed mother, child, and household, those that have been found to not be correlated with TLU per capita but to be significantly associated with either the HFIAS or DDS were included in the HFIAS and/or DDS models. These independent variables were maternal education, mother’s age, a dummy for the monthly age and sex of the surveyed child, the number of months for which a respondent’s household could rely on their own produced rice in the last 12 months, the frequency of antenatal check-ups in the last 12 months, a dummy for obtaining nutritional information from CNAs and/or health facilities, and the paternal financial support regarding children’s food. Given the significant positive correlation between TLU per capita and the wealth index (principal component score estimated from the possession of multiple durable goods), the wealth index was not used as an explanatory variable in order to avoid the well-known multicollinearity problem. The means and standard deviations for the dependent variables are presented in Table 1.

All analyses were performed using Stata version 17.0.

### 2.4. Ethical Considerations

This research was approved by the Ministry of Public Health of Madagascar and the Institutional Review Board of Oklahoma State University, Stillwater, OK, USA. Respondents’ personal information and information provided by the respondents were kept confidential, and interviews took place only after obtaining the respondents’ consent and in the presence of a witness.

## 3. Results

Employing Coates et al.’s [24] formula, among the 344 respondent households, 189 (54.9%) fell into the severely food-insecure category, 117 (34.0%) into the moderately food-insecure category, 14 (4.1%) into the mildly food-insecure category, and only 24 (7.0%) into the food-secure category, suggesting that approximately every nine in ten households suffered from severe or moderate food insecurity.

**Figure 1:**
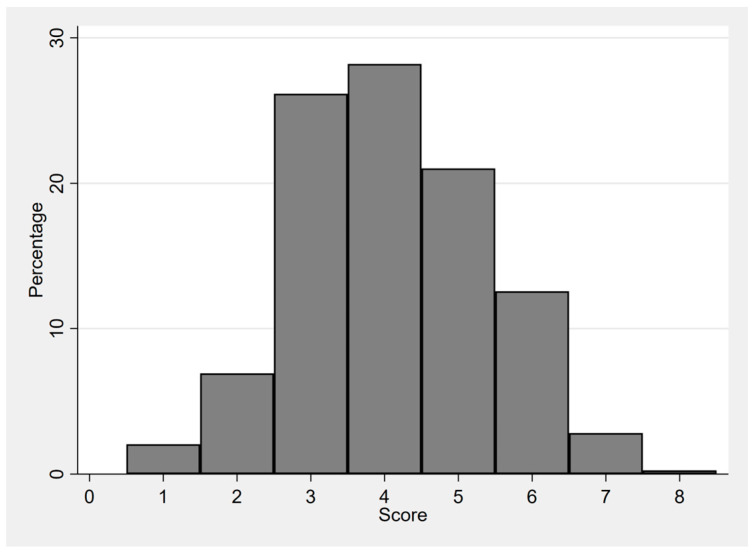
Distribution of scores for dietary diversity.

**Figure 2:**
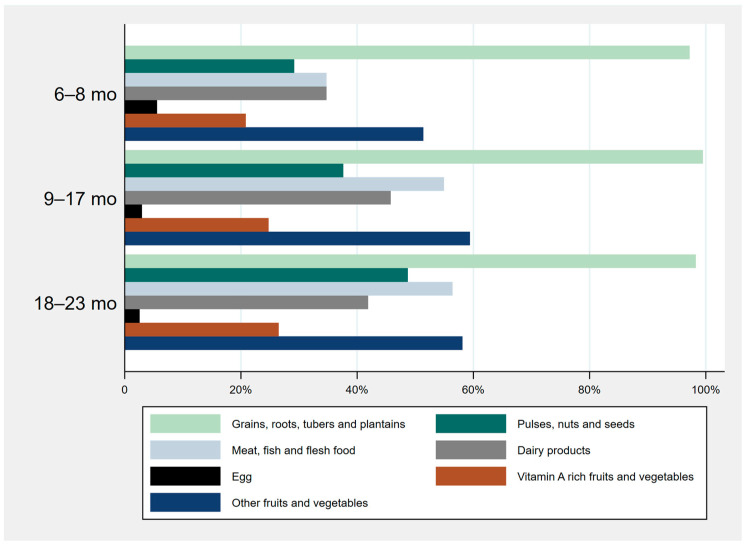
Food group consumption per age section.

The mean DDS was 4.090 (±1.312) out of 8. A total of 218 children (63.4%) did not meet the minimum dietary diversity (Figure 1). Considering that 91.9% of the surveyed children were breastfed on the day previous to the survey, every two out of three children consumed only two to three other complementary food groups to complete their diet. Almost all of them had eaten cereals and/or tubers the previous day. The children’s diets were mainly composed of rice and only one other food group (Figure 2). Although more than half of the surveyed households owned poultry, there was a particularly low consumption of eggs among all ages (3.5% for the entire region).

**Figure 3:**
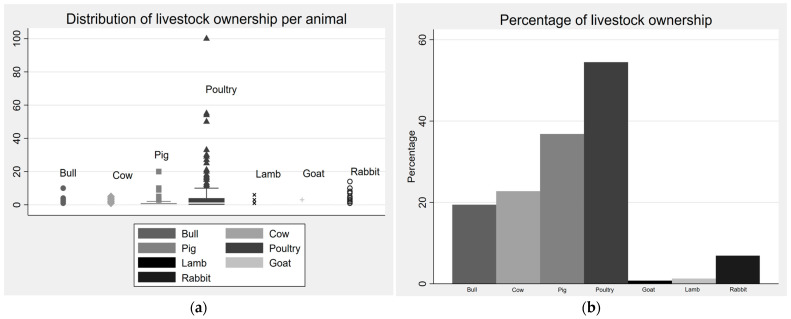
Livestock ownership in the region of Vakinankaratra.

Figure 3 shows the distribution of livestock ownership in the surveyed regions. Among the 344 surveyed households, 279 (81.1%) practiced livestock rearing for some sort. Poultry (54.1%) was the most common livestock, followed by pigs (37.8%), cows (22.4%), bulls (19.8%), rabbits (7.0%), lambs (1.2%), and goats (0.3%). Only 65.4% of the respondents owned cows or bulls. The average number of animals for each livestock-owning household was 7.6 chickens, 1.6 pigs, 1.4 cows, 1.6 bulls, 4.3 rabbits, 2.8 lambs, and 3.0 cows.

As the correlation coefficient (ρ) between the disturbance terms in the bivariate ordered probit model was not significant at the 5% level (ρ = 0.004, *p* = 0.953), the coefficients of the two equations for the HFIAS and DDS were estimated separately using the ordered probit model.

The estimation results from the ordered probit model showed that higher household TLU per capita was associated with a better food security status (*β* = −0.720, *p* = 0.049) and better DDSs for children (*β* = 0.356, *p* = 0.031) (Table 2). A negative association was also observed between the HFIAS and the number of months that households could depend on their own rice production (*β* = −0.041, *p* = 0.013), whereas the DDS was not significantly related to this variable. Higher DDS was associated with more antenatal care visits (*β* = 0.080, *p* = 0.003) and when mother received nutrition information from health facilities and/or CNAs (*β* = 0.345, *p* = 0.012). Common associations were observed between mothers’ education and fathers’ financial support for children’s nutrition, emphasizing the importance of parental qualities in food security and improvement of children’s dietary diversity.

The estimation result for the marginal effects of TLU per capita for HFIAS was −0.260 for severe food insecurity (Table 3), showing that, with keeping all variables constant, increases of ten poultry/rabbits, one pig, or a cow could decrease the probability of severe food insecurity from 59.4% by 2.6%, 5.2%, and 18.2%, respectively. Therefore, the effects of reducing severe food insecurity by raising livestock were not small compared to those of mothers’ education (marginal effect was −0.038), consumption of own rice production (marginal effect was −0.015). 

An increase in the TLU per capita by one unit also could increase the probability of children having a DDS of 6 (4.7%), 7 (5.8%), or 8 (2.2%) (Table 4), meaning that the probability of children to meet the minimum dietary diversity will rise by approximately 12.7% point with an increase of one unit of TLU per capita. In other words, increases of ten poultry/rabbits, one pig, and one head of cattle could be expected to increase the probability of clearing the criteria of the five-point DDS from 16.4% by 1.3%, 2.5%, and 8.9%, respectively. The predicted probability of achieving good dietary scores of 5 and above was found to increase by 2.2% with a one-year increase in mothers’ education, by 2.9% with an increase of one antenatal check, by 12.1% when getting information from health facilities and/or CNAs, and by 15.3–16.2% with fathers’ financial support for children’s food.

## 4. Discussion

This study demonstrated the importance of livestock in improving food security and children’s dietary patterns in the rural region of Vakinankaratra, Madagascar. The TLU per capita was negatively associated with HFIAS and positively associated with DDS, indicating that farmers possessing livestock were more likely to be food secure and able to feed their children a more diverse diet. While there is limited research on livestock relating to children’s diets, several studies, including Rakotonirina et al. [9], Murendo et al. [10], and Dangura et al. [51], have demonstrated the positive association between livestock ownership and dietary diversity. The current study is the first to demonstrate this in Vakinankaratra.

Livestock and agricultural production represent the backbone of the economy for smallholder farmers from rural areas such as the Vakinankaratra Region. The main role of livestock in sustaining small farmers’ livelihoods has already been demonstrated in several studies conducted in different countries in Africa and Asia. According to Chen et al. [52], the most common pathways linking livestock production to nutrition include food production, income generation, and women’s empowerment. In addition to supporting crop production by producing manure and work labor [53,54], livestock also provides animal-source food for both household members and the local markets [55,56,57]. Furthermore, their by-products can be revalorized. Ahmed et al. [58] demonstrated the importance of livestock in resource limited areas, as livestock can potentially provide insurance in cases of emergencies, food shortages, or during the lean seasons. Other investigations [59,60,61,62,63,64,65] have shown that the pathway from livestock to food security and nutrition promotes women’s empowerment by enabling women to manage and make accounting decisions for small livestock and promoting income generation. In Madagascar, initial studies have demonstrated that promoting women’s decision making at the household level can improve their engagement in better farming practices [66] while alleviating their socio-economic conditions, and ultimately could prevent their children from malnutrition [21]. However, further studies are needed to better understand the links between women empowerment, livestock ownership and child nutrition. 

Although no data concerning the potential origins of animal-source foods, —whether they came from the farm or elsewhere, —were collected during the survey, this study revealed the great importance of increasing livestock farming size for the promotion of dietary diversity among children, as most farmers manage small-scale productions. The World Bank [67] stated that several reasons may explain this lower scale of livestock farming, including const constraints of raising livestock and the recurrence of uncontrollable incidences, such as diseases, natural disasters, and theft. Nevertheless, farmers should consider the benefits that arise from livestock including the promotion of child nutrition, especially in the Vakinankaratra region.

Receiving nutrition information from community nutrition agencies or health facilities has increased the probability of achieving minimum dietary diversity in the Vakinankaratra region. Children of mothers who acknowledged receiving nutrition information and counseling from community nutrition agents achieved better dietary diversity scores than those who did not [68]. Other studies [69,70,71,72,73] also confirmed the importance of nutrition information messaging to mothers on dietary diversity among children. Since encouraging mothers to attend CNCs’ meetings regularly may help children achieve higher dietary scores to some extent, this study recommends the continuing support towards CNCs in their services, particularly nutrition counseling for mothers.

A more diversified diet was observed among children whose mothers visited health facilities during pregnancy and had periodic antenatal checks. Similar results have been reported in other countries [74,75,76,77], showing better nutrition outcomes among mothers with health-seeking behaviors. Madagascar is a low-income country with a poor health infrastructure, especially in rural areas. Mothers showing health-seeking behaviors by visiting health centers to check for pregnancy and other isues related to the child are more likely to give more diverse diets [78]. The importance of antenatal checks during pregnancy has been supported by many studies conducted in Africa [68,73]. Studies conducted in South Asia have reported similar findings, especially regarding the association between maternal antenatal checkups and children’s dietary profiles [79,80]. Many factors can explain why mothers rarely visit health facilities during pregnancy, including socioeconomic reasons, the mother’s marital status, past birth experiences, low maternal educational levels, pregnancy neglect, religion and cultural beliefs, women’s empowerment and domestic violence, a lack of support from men, low family income, and resource issues including a lack of infrastructure and medical staff in health centers [81,82,83,84,85]. Assisting mothers to attend antenatal checks and addressing barriers that prevent them from going to health facilities during pregnancy may, therefore, improve their children’s nutrition. 

This study also allowed us to recognize the importance of paternal financial support for children’s food and their dietary profiles, as well as contributing to household food security, as previously reported in similar contexts [68,86]. In many African countries, including Madagascar, the father represents the main household breadwinner and manages the household finances. In many contexts, women have limited ability to use money to buy food for their children without consulting men or obtaining approval [87,88,89,90]. Consequently, paternal willingness to provide support for children’s food is crucial in such contexts [91,92]. At the household level, fathers are concerned about the entire household’s access to daily food supplies, particularly the children’s food [93,94]. Their involvement in providing financial support for nutrition arises from their acknowledgement of the importance of a good diet, which in turn contributes to better food and nutrition security [86,95]. Similar results were obtained in different studies conducted in Madagascar and Ethiopia, which used qualitative approaches to identify the role of paternal involvement in childcare and feeding practices [96,97].

Finally, this study allowed us to better understand the different dimensions to consider for more effective interventions on food insecurity and child undernutrition [98] in rural Madagascar. Smallholder farmers may benefit from initiatives that reinforce rural activities, notably livestock farming so that households may increase food accessibility, especially during the lean season. Also, interventions that aim to improve children’s dietary pattern need to encourage maternal health-seeking behaviors during and after pregnancy and strengthen nutrition counseling at community level. Similar conclusions were drawn in other studies including Ruel et al. [65], Sharma et al. [99], and Christiaensen et al. [100]. Policymakers should, therefore, address the target of their interventions and their expectations of the respective results more carefully, regardless of whether it is for enhancing household food security, improving children’s nutrition through complementary feeding practices, or both.

### Limitations and Implications of the Study

To the best of our knowledge, this study is the first to confirm the importance of livestock ownership on food security and child diet in rural areas of Madagascar. Future studies can build on the results of this study to deepen the understanding of the links between livestock rearing, food security, and child nutrition in rural Madagascar and other similar contexts. Another strength of this study is also its geographical coverage with 43 fokontany in the Vakinankaratra region, capturing a range of different household contexts.

However, this study has some limitations. First, as surveys were conducted from mid-March to mid-April, some respondents had finished harvesting, while others were still waiting for or working on the rice harvest. This dissimilarity in the harvest calendar could have biased the results concerning food consumption and the food security experiences of households, as many of them had mobilized funds to support the harvest, while others started to use the harvested products.

Also, it was assumed that there was a significant correlation between TLU per capita and non-livestock assets or income levels. Therefore, it is possible that the effects of non-livestock assets and income levels were included in our estimates of the effects of livestock production on the nutritional status of children. However, due to difficulties in obtaining accurate information on household income from maternal respondents, this variable could not be included in the analysis. In addition, due to the confounding nature of non-livestock assets in relation to TLU per capita, they were omitted from the final estimation model. Given the volatility of monthly income and the fact that assets such as durable goods do not directly affect food intake, the conclusion of this study—that raising livestock, which is expected to increase households’ consumption of animal protein or provide income from selling products, has a positive effect on children’s nutritional status—is plausible. However, panel data or more well-designed analyses should be used to confirm this finding further.

Lastly, while main role for livestock products, as well as for the perception and knowledge of mothers concerning the consumption or introduction of livestock-derived products in children’s diets, was not collected by this study, further investigations are needed to explore the extent to which livestock may influence food security and child nutrition in the region.

This study suggests that policy interventions should be as specific as possible and respond to targeted problems before being implemented at the community level in order to be effective. In a highly productive region such as Vakinankaratra, any intention to reduce food insecurity need to include both crop and livestock production adequate to the local context [101]. Moreover, improvement in household food security does not automatically imply an improvement in the quality of children’s diets in the Vakinankaratra region. Rather, enhancing children’s diets require, in addition, more focus on nutrition-related interventions such as improvements in quality and access of services provided in each community nutrition centers.

## 5. Conclusions

In the Vakinankaratra region of Madagascar, livestock rearing influences household food security status and dietary diversity among children to some extent. While farmers raising a larger number of livestock are likely to be food-secure, children can also acquire a more varied diet through the benefits that arise from owning livestock. Additionally, the dietary diversity of children was found to be more strongly associated with mothers’ health-seeking behaviors and gender equality in childcare.

This research can help policy makers and various stakeholders direct efficient interventions regarding food security and nutrition, especially with respect to the targets of these interventions. The main objectives must be defined prior to each intervention and targeted at either improving the nutritional patterns of children or enhancing livelihoods and ensuring the resilience of households at the same time. Poverty remains one of the basic causes of food insecurity and malnutrition, so implementation of any intervention must consider the local context and the resources available for overcoming chronic poverty. As in the case of Madagascar, many interventions have been implemented to fight undernutrition among children but only a few have focused on education-related programs communicating the importance of raising livestock for household food security and children’s nutritional intake. Furthermore, enlightening mothers and their spouses about the implications of raising livestock for children’s nutritional status, in addition to promoting maternal health-seeking behaviors, is essential.

## Figures and Tables

**Table 1:** Characteristics of the study population, n = 344.

	Mean (SD) or Frequency (%)
Child characteristics	
Dummy for age	
6–8 months (reference) 9–17 months 18–23 months	0.183 (0.387) 0.523 (0.500) 0.294 (0.456)
Dummy for gender	
Male (reference) Female	0.515 (0.501) 0.485 (0.501)
Maternal characteristics	
Age (years)	27.390 (6.682)
Formal education (years)	5.828 (3.093)
Household characteristics	
TLU * per capita	0.133 (0.188)
Number of months household could depend on its own produced rice in the last 12 months (months)	4.004 (3.215)
Other characteristics	
Number of antenatal check-ups in the last 12 months (times)	3.968 (1.400)
Dummy for getting nutritional information from CNAs ^+^ and/or health facilities (yes = 1, otherwise = 0)	0.686 (0.465)
Dummy for paternal financial support for child’s food	
Seldom (reference) Sometimes All the time	0.076 (0.265) 0.328 (0.470) 0.596 (0.491)

* TLU: tropical livestock unit. ^+^ CNA: community nutrition agent.

**Table 2:** Determinants of HFIAS scores and DDSs from a multivariate ordered probit analysis.

Multivariate Ordered Probit Model	HFIAS	DDS ^+^
Variables	Coefficients	Coefficients
Maternal characteristics		
Age (years)	−0.006 (0.006)	0.015 (0.012)
Formal education (years)	−0.105 (0.020) ***	0.062 (0.020) ***
Household characteristics		
TLU per capita	−0.720 (0.365) **	0.356 (0.165) **
Dependence on own produced rice (months)	−0.041 (0.017) **	0.021 (0.022)
Other characteristics		
Antenatal check-ups (months)	−0.014 (0.041)	0.080 (0.026) ***
Dummy for getting nutritional information (yes = 1, no = 0)	−0.070 (0.128)	0.345 (0.139) **
Dummy for paternal financial support for child’s food		
Seldom (reference)		
Sometimes	−0.403 (0.329)	0.495 (0.145) ***
All the time	−0.618 (0.311) **	0.470 (0.191) **
Child characteristics		
Dummy for child’s age		
6–8 months (reference)		
9–17 months		0.325 (0.231)
18–23 months		0.187 (0.232)
Observations	344	344

Robust standard errors are shown in parentheses. *** *p* < 0.01, ** *p* < 0.05. ^+^: As only one child had a DDS of 8, they were included along with the children with DDSs of 7 in the above estimation.

**Table 3:** Marginal effects for the HFIAS.

	Severely Insecure	Moderately Insecure	Mildly Insecure	Secure
Maternal characteristics				
Age (years)	−0.002 (0.002)	0.001 (0.001)	0.000 (0.000)	0.001 (0.001)
Formal education (years)	−0.038 (0.007) ***	0.020 (0.003) ***	0.005 (0.002) ***	0.013 (0.002) ***
Household characteristics				
TLU per capita	−0.260 (0.129) **	0.137 (0.068) **	0.036 (0.014) **	0.087 (0.049) *
Dependence on own produced rice (months)	−0.015 (0.006) ***	0.008 (0.003) **	0.002 (0.001) **	0.005 (0.002) ***
Other characteristics				
Antenatal check-ups (months)	−0.005 (0.015)	0.003 (0.008)	0.001 (0.002)	0.002 (0.005)
Dummy for getting nutritional information (yes = 1, no = 0)	−0.025 (0.046)	0.013 (0.024)	0.003 (0.006)	0.009 (0.015)
Dummy for paternal financial support for child’s food				
Seldom (reference)				
Sometimes	−0.136 (0.104)	0.089 (0.073)	0.016 (0.012)	0.031 (0.021)
All the time	−0.215 (0.097) **	0.131 (0.069) *	0.027 (0.013) **	0.057 (0.018) ***
Observations	344	344	344	344

Robust standard errors in parentheses. *** *p* < 0.01, ** *p* < 0.05, * *p* < 0.1.

**Table 4:** Marginal effects for DDS.

	DDS = 5	DDS = 6	DDS = 7 and 8
Maternal characteristics			
Age (years)	0.002 (0.001)	0.002 (0.002)	0.001 (0.001)
Formal education (years)	0.008 (0.003) ***	0.010 (0.003) ***	0.004 (0.002) **
Household characteristics			
TLU per capita	0.047 (0.023) **	0.058 (0.028) **	0.022 (0.011) *
Dependence on own produced rice (months)	0.003 (0.003)	0.003 (0.004)	0.001 (0.001)
Other characteristics			
Antenatal check-ups (months)	0.011 (0.004) ***	0.013 (0.004) **	0.005 (0.002) **
Dummy for getting nutritional information (yes = 1, no = 0)	0.049 (0.02) **	0.054 (0.024) **	0.018 (0.007) ***
Dummy for paternal financial support for child’s food			
Seldom (reference)			
Sometimes	0.072 (0.024) ***	0.069 (0.020) ***	0.021 (0.009) **
All the time	0.069 (0.031) **	0.065 (0.022) ***	0.019 (0.008) **
Child characteristics			
Dummy for child’s age			
6–8 months (reference)			
9–17 months	0.045 (0.031)	0.051 (0.037)	0.051 (0.037)
18–23 months	0.027 (0.034)	0.028 (0.035)	0.028 (0.035)
Observations	344	344	344

Robust standard errors are shown in parentheses. *** *p* < 0.01, ** *p* < 0.05, * *p* < 0.1.

## Data Availability

Due to privacy and ethical consideration, the research data cannot be shared. For any requests, please address to the authors.
